# Supplementary material for: Ratiometric Near-Infrared Fluorescence Liposome Nanoprobe for H2S Detection In Vivo
Source: Molecules. 2023 Feb 16;28(4):1898. doi: 10.3390/molecules28041898 (PMC9961796; doi:10.3390/molecules28041898)
Supplement: Supplementary file 1 [file molecules-28-01898-s001.zip › molecules-2198724-supplementary.pdf]

Ratiometric near-infrared fluorescence liposome nanoprobe for H_2S detection in vivo

Luyan Wu[1,*,+,] , Yili Liu [2,+], Junya Zhang [2], Yinxing Miao [2] and Ruibing An[3,*]

[1] Jiangsu Key Laboratory for Biosensors, Key Laboratory for Organic Electronics and Information Displays, Institute of Advanced Materials (IAM), Jiangsu National Synergetic Innovation Center for Advanced Mate-rials (SICAM), Nanjing University of Posts and Telecommunications, Nanjing, 210023, China

[2] State Key Laboratory of Analytical Chemistry for Life Science, Chemistry and Biomedicine Innovation Center (ChemBIC), School of Chemistry and Chemical Engineering, Nanjing University, Nanjing, 210033, China

[3] Institute of Optical Functional Materials for Biomedical Imaging, School of Chemistry and Pharmaceutical Engineering, Shandong First Medical University & Shandong Academy of Medical Science, Taian, Shandong 271016, P. R. China

[*]Correspondence: iamlywu@njupt.edu.cn; anruibing@sdfmu.edu.cn

[+] These authors contributed equally to this work.

Table of Contents

		Page
1	Fig. S1-8	S2-7
2	Experimental Section	S8

Figure S1-8

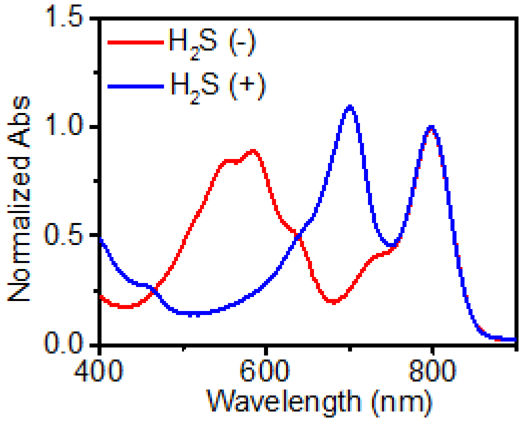

Figure S1. UV-Vis-NIR absorption spectra. UV-Vis-NIR absorption spectra of HS-CG before (-) and after (+) reaction with NaHS (500 μM) for 6 min in PBS buffer.

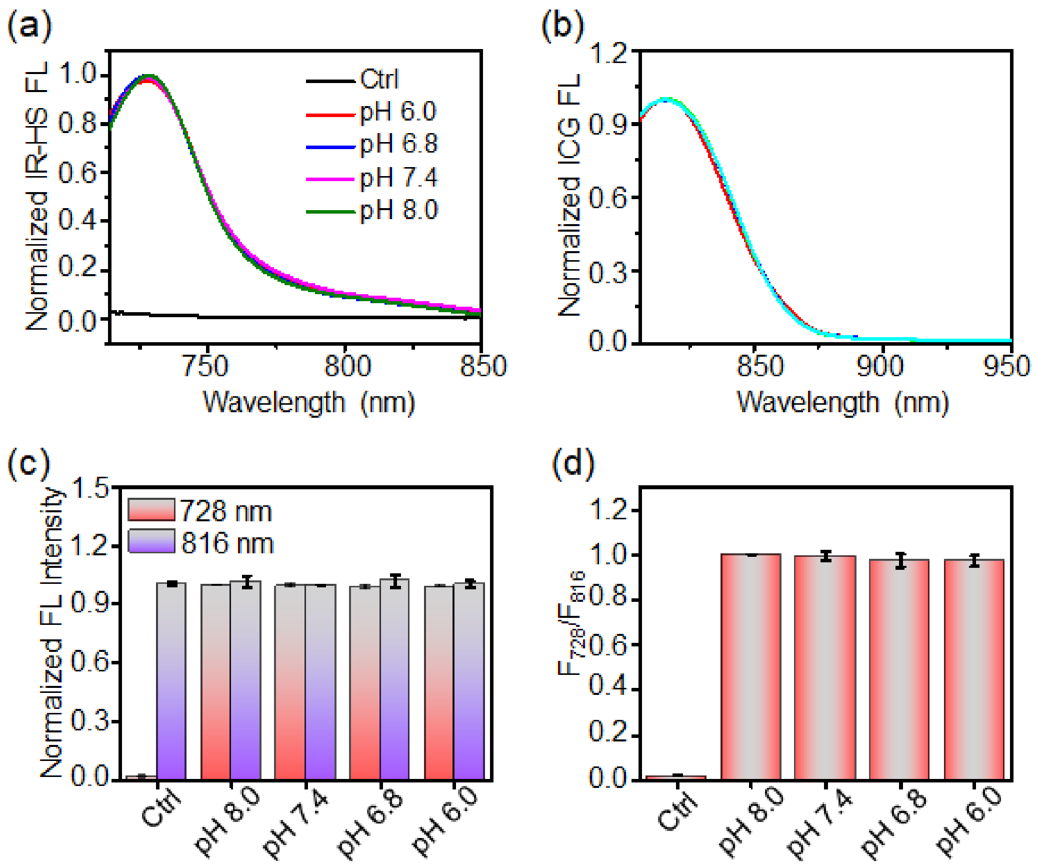

Figure S2. Response of HS-CG to H_2S in PBS with different pH. Fluorescence spectra of (a) IR-HS and (b) ICG within HS-CG, and (c) intensities at 728 and 816 nm and (d) ratiometric fluorescence (F_{728}/F_{816}) of HS-CG after reaction with NaHS (500 μM) in PBS buffer under indicated pH for 24 h. Fluorescence of HS-CG in PBS buffer (pH 7.4) as control group (Ctrl). Values are mean ± s.d. (n = 3).

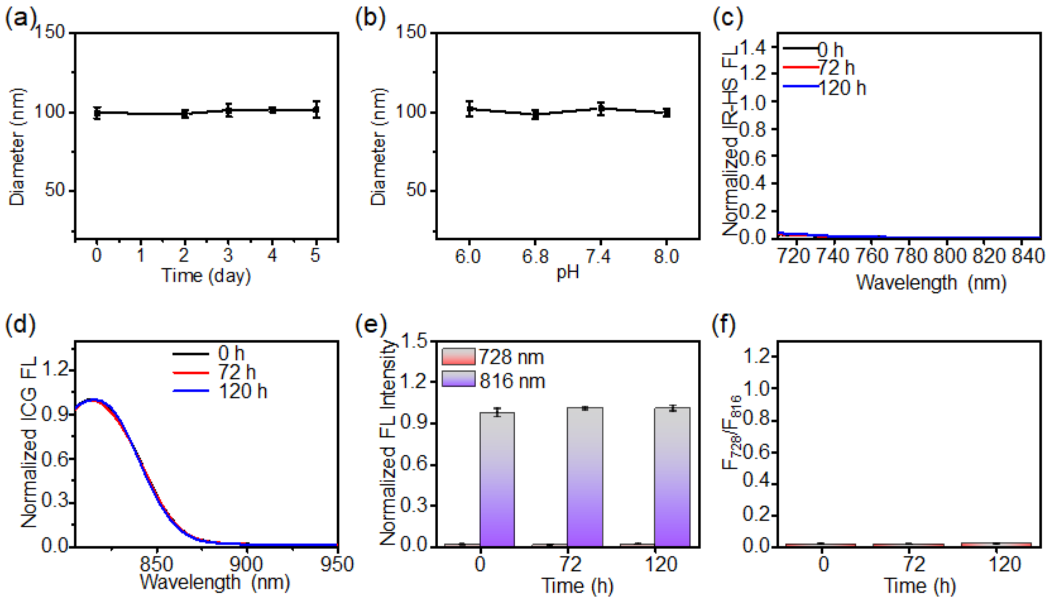

Figure S3. Evaluation of the stability of HS-CG. (a) Comparison of the hydrodynamic size of HS-CG following incubation in PBS buffer (pH 7.4) for 5 days. (b) Comparison of the hydrodynamic size of HS-CG following incubation in PBS buffer under different pH for 24 h. Comparison of fluorescence spectra of (c) IR-HS and (d) ICG within HS-CG following incubation in PBS buffer (1×, pH 7.4) for 5 days. (e) Comparison of fluorescence intensities at 728 and 816 nm of HS-CG following incubation in PBS buffer (1×, pH 7.4). (f) Comparison of fluorescence ratio (F_{728}/F_{816}) at 728 and 816 nm of HS-CG following incubation in PBS buffer (1×, pH 7.4). Data denote mean ± standard deviation (s.d.) (n = 3).

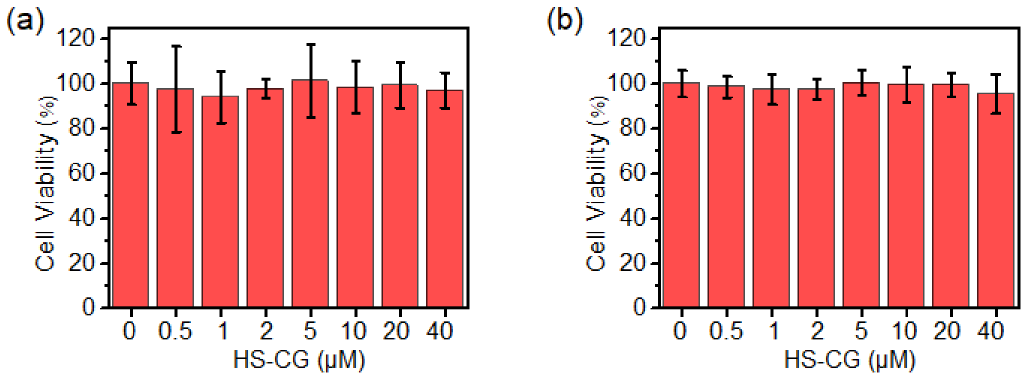

Figure S4. Evaluation of the cytotoxicity. Cytotoxicity of HS-CG against (a) HCT116 colorectal cancer cells and (b) human embryonic kidney cells (HEK293 cells). Cells were incubated with indicated concentration of HS-CG (based on IR-HS and ICG) for 24 h, and the cell viability was tested by MTT assay.

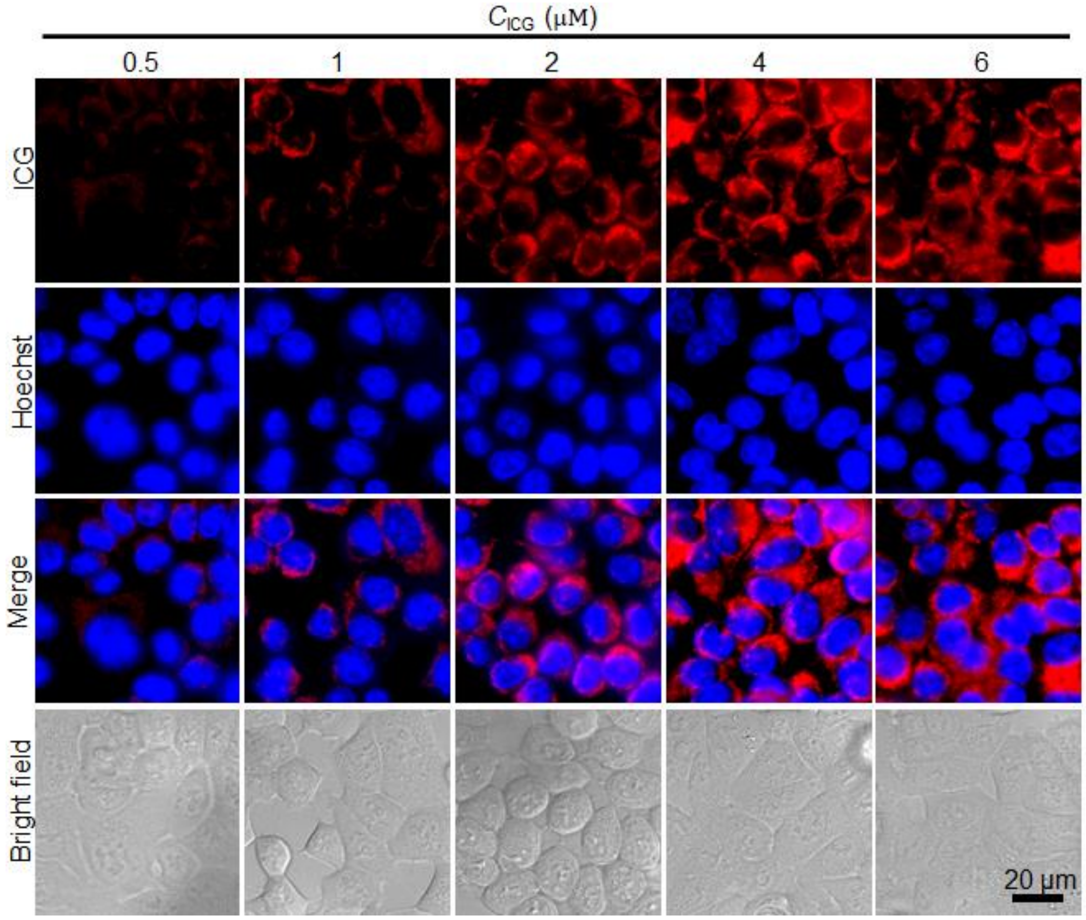

Figure S5. Optimization of incubation concentration of HS-CG in HCT116 cells. Fluorescence images of HCT116 cells incubated with different concentration of HS-CG (0.5, 1, 2, 4, 6 μM ICG) for 3 h.

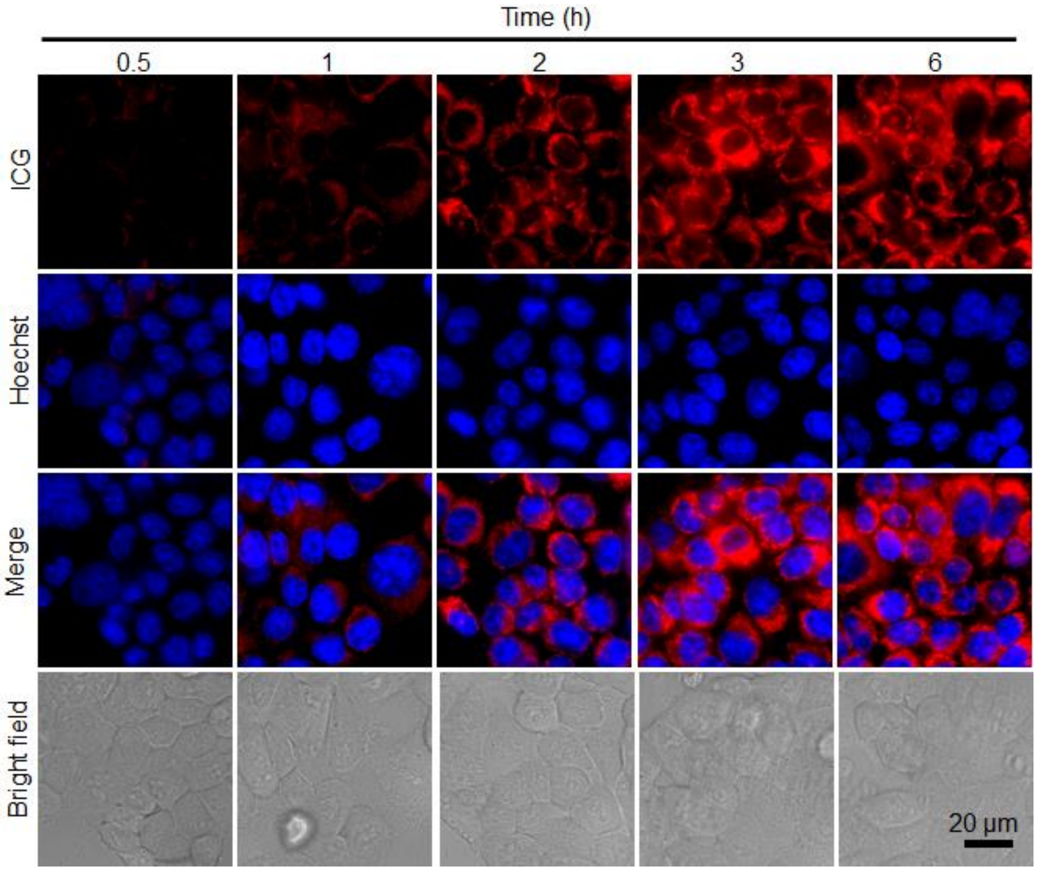

Figure S6. Optimization of incubation time of HS-CG in HCT116 cells. Fluorescence images of HCT116 cells incubated with HS-CG (4 μM ICG) for different time (0.5, 1, 2, 3, 6 h).

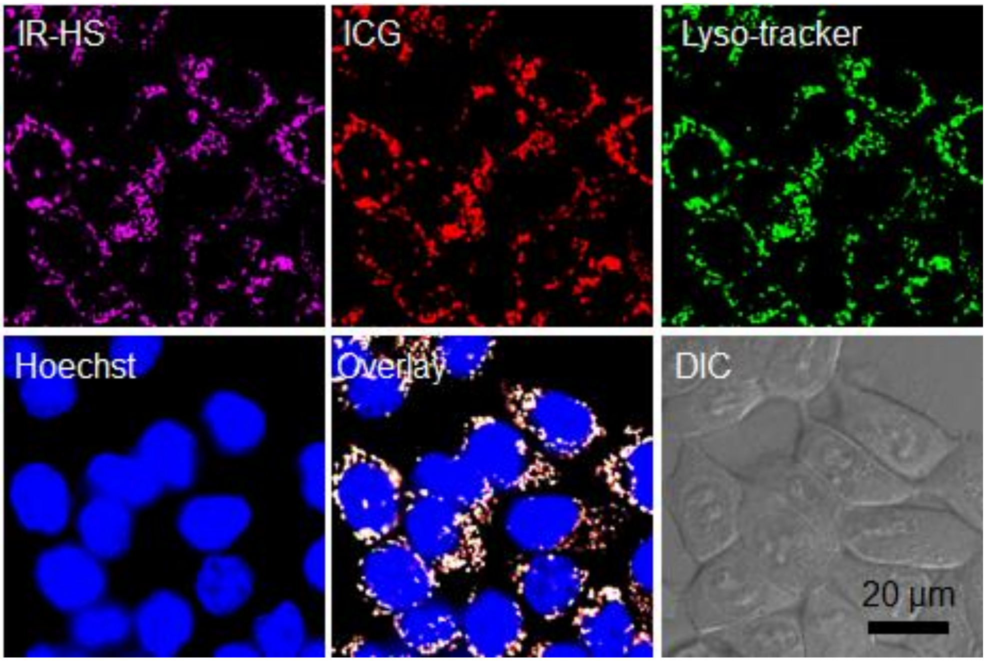

Figure S7. Colocalization study of HCT116 cells incubating with HS-CG and Lyso-tracker. Fluorescence images of HCT116 cells incubated with HS-CG, Lyso-tracker red and Hoechst. HCT116 cells were pretreated with HS-CG (4/4 μM IR-HS/ICG, 3 h) and co-stained with Lyso-tracker red (2 μM, 20 min).

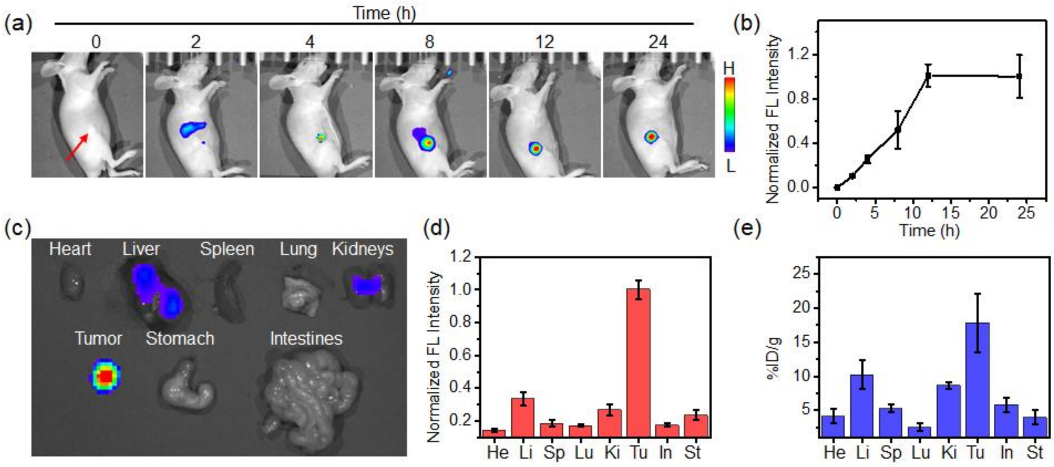

Figure S8. Fluorescence imaging of HS-CG in vivo and ex vivo, and biodistribution of HS-CG. Time-dependent fluorescence imaging (a) and intensity (b) for ICG in HCT116 tumor-bearing mice post i.v. injection of HS-CG (40/40 μM IR-HS/ICG, 200 μL). Red arrow indicates the location of tumor in the mouse. Representative ex vivo fluorescence images (c) and intensities (d) for ICG of main organs (e.g., heart (He), liver (Li), spleen (Sp), lung (Lu), kidneys (Ki), tumor (Tu), stomach (St) and intestines (In)) resected from HCT116 tumors-bearing mice with i.v. injection of HS-CG (40/40 μM IR-HS/ICG, 200 μL). (e) Biodistribution of HS-CG (40/40 μM IR-HS/ICG, 200 μL) at 12 h post i.v. injection into HCT116 tumor-bearing mice.

Experimental Section

Methods

LOD calculations for HS-CG towards H_2S

Fluorescence intensity ratio (F_{728}/F_{816}) was applied for the calculation the detection limit according to the formula ($3\delta/k$), where δ is the standard deviation of F_{728}/F_{816} of 11 blank signal; k is the slope between the F_{728}/F_{816} versus H_2S concentration.

In order to check the instrumental noise, we also re-measured the fluorescence at 728 and 816 nm of 11 blank buffer using the same experimental conditions for HS-CG. Normalized fluorescence at 728 nm of 11 blank signal were 0.01925, 0.01823, 0.01756, 0.01978, 0.01856, 0.01989, 0.01732, 0.01689, 0.01789, 0.01896 and 0.01987. Normalized fluorescence at 816 nm of 11 blank signal were 1, 1.003, 1.105, 1.045, 1.112, 1.105, 0.9965, 0.987, 0.9987, 1.023 and 1.109. The standard deviation (δ) of 11 blank measurements of fluorescence ratio (F_{728}/F_{816}) were measured to be 0.000982 for HS-CG. Therefore, the detection limits (3δ/k) were calculated to be ~0.13 μM for HS-CG. These results suggested that HS-CG possessed high sensitivity for H2S.
